# Association between functional polymorphisms in *IL‐33*/*ST2* pathway and risk of osteosarcoma

**DOI:** 10.1111/jcmm.13653

**Published:** 2018-05-23

**Authors:** Jun‐Li Wang, Jia Liu, Ke‐Gong Xie, Chang‐Gong Lan, Lu Lu, Yu‐Jin Tang

**Affiliations:** ^1^ Department of Clinical Laboratory The Affiliated Hospital of Youjiang Medical University for Nationalities Baise Guangxi China; ^2^ Department of Orthopedic Surgery The Affiliated Hospital of Youjiang Medical University for Nationalities Baise Guangxi China

**Keywords:** *IL‐33*, osteosarcoma, plasma, polymorphism, *ST2*

## Abstract

Interleukin (IL)‐33/ST2 pathway plays crucial roles in tumour growth and metastasis. The aim of this study was to investigate the association of two functional polymorphisms (*IL‐33* rs7025417 and *ST2* rs3821204) with osteosarcoma (OS) risk. The rs7025417 and rs3821204 were genotyped by Taqman assay. *IL‐33* mRNA and protein levels were measured by real‐time PCR or enzyme‐linked immunosorbent assay. The luciferase activity was measured by a dual luciferase reporter gene assay. The allele‐specific transcription factor binding for rs7025417 was examined by ChIP‐seq. The *IL‐33* rs7025417 CC genotype was significantly associated with a decreased risk of OS (CC vs TT: OR = 0.59, 95% CI, 0.41‐0.85; recessive model: OR = 0.68, 95% CI, 0.49‐0.94; C vs T: OR = 0.76, 95% CI, 0.63‐0.91). Combined analysis showed that the *IL‐33* rs7025417CT/CC‐*ST2* rs3821204CG/CC and the *IL‐33* rs7025417CT/CC‐*ST2* rs3821204GG genotypes also had a decreased risk of OS. *IL‐33* mRNA and protein levels in OS patients were significantly higher than controls. Patients with the rs7025417 CC genotype exhibited lower levels of *IL‐33* (*P* = .03). The rs7025417 C allele presented a lower transcriptional activity by disrupting the binding site to c‐Myb (*P *<* *.01). Moreover, the rs3821204 G/C influences the transcriptional activity and *ST2* mRNA expression by altering the binding site of miR‐202‐3p. These findings suggest that the rs7025417 and rs3821204 may have a combined effect to protect against the development of OS by decreasing the expression levels of *IL‐33* or *ST2*.

## 1 INTRODUCTION

Osteosarcoma (OS) is the most common primary malignant neoplasm in bone and accounts for about 60% of all cancer‐related death in children and adolescents.^1^, ^2^, ^3^ Despite improvements of therapeutic strategies, the overall 5‐year survival rate remains 60%‐70%.^4^, ^5^, ^6^, ^7^ Treatment often fails because of recurrent OS and/or metastasis, especially pulmonary metastasis that is the major cause of death.^5^, ^7^ Therefore, it is vital to better understand the biological aetiology underlying the development and progression of OS. Previous studies have demonstrated some risk factors for the carcinogenesis of OS such as radiation and chemicals exposure.^8^, ^9^ However, not all individuals develop OS when exposed to these risk factors, indicating that genetic factors may be involved in the development of OS. Our previous work showed that interleukin (*IL*)*‐12A* rs568408 GA and GA/AA genotpyes, *IL‐12B* rs3212227 CC and AC/CC genotypes, and *IL‐16* rs11556218 TG genotype may confer the susceptibility to OS risk.^10^, ^11^ Besides these cytokines' polymorphisms, *IL‐1B*‐31CC and ‐511TT were reported to be protective factors against OS.^12^

IL‐33, a member of the IL‐1 family, is secreted by a wide variety of cell types, including fibroblasts, mast cells, dendritic cells, macrophages, osteoblasts, endothelial cells and epithelial cells.^13^ Binding of IL‐33 to its receptor ST2 induces the activation of NF‐κB and Th‐2 pro‐inflammatory cytokines, such as IL‐4, IL‐5 and IL‐13,^14^, ^15^ which play important roles in the development of OS.^16^, ^17^ Epithelial‐derived IL‐33 can promote tumorigenesis and metastasis both in vivo and in vitro.^18^, ^19^, ^20^ These findings implied that IL‐33 may be tumour‐associated pro‐inflammatory cytokine.

*IL‐33*, located on chromosome 9p24.1 in the human genome, contains several single nucleotide polymorphisms (SNPs) within the full sequence. Most of them, however, are located in the intron of *IL‐33* and have no function. In this study, we focused on a functional polymorphism rs7025417 that are located in the promoter of *IL‐33* (−1611 bp from the transcription start site), with the different genotypes affecting the levels of circulating IL‐33.^21^ Because *IL‐33* exerts functions via ST2, another SNP rs3821204 was selected, which was located within the 3′ untranslated region (UTR) of *ST2* mRNA. The rs3821204 can disrupt the binding site of miR‐202‐3p and finally affect plasma‐soluble ST2 levels.^22^ To date, no study has reported on the association of the 2 functional SNPs with OS risk. The aim of this study was to investigate whether the 2 SNPs influenced the susceptibility to OS in a Chinese population. Moreover, whether the SNPs influence on *IL‐33* expression levels and transcriptional activities were also examined.

## 2 MATERIALS AND METHODS

### 2.1 Ethics, consent and permissions

The hospital‐based case‐control study was approved by the Review Boards of Affiliated Hospital of Youjiang Medical College for Nationalities. All individuals agreed to participate in the study and provided informed consent.

### 2.2 Consent to publish

The participants signed the consent to publish the data.

### 2.3 Study population

Totally, 402 OS patients and 572 controls were selected from the Affiliated Hospital of Youjiang Medical College for Nationalities and the West China Hospital between January 2008 and August 2016. Detailed information of the study participants was described in our previous study.^11^ Briefly, OS patients were diagnosed by histological examination. The following information was collected: age of diagnosis, gender, family history of cancer, tumour location and metastasis status. Patients who had a history of familial cancer were excluded from this study. The healthy controls were recruited from the same hospital with the following selection criteria: no cardiovascular diseases, hypertension, diabetes mellitus and other inflammatory diseases; no history of any cancer and no family history of any cancer. All the participants in this study were unrelated Han Chinese.

### 2.4 SNPs selection

It is well known that SNPs in the non‐coding region of genes contribute to the susceptibility to OS. In this study, we selected SNPs according to the following criteria: (i) SNPs located in the non‐coding region of *IL‐33* or *ST2*; (ii) functional SNPs with known biological mechanism based on published literatures. Finally, 2 SNPs (ie, rs7025417 and rs3821204) were identified. The rs7025417 in the promoter of *IL‐33* affects the levels of circulating IL‐33 by influencing the reporter activity^21^ and the rs3821204 in the 3′ UTR of *ST2* affects soluble ST2 levels by altering the binding site of miR‐202‐3p.^22^

### 2.5 Genotyping

Fasting venous blood was obtained from peripheral vein of each participant. After centrifugation at 1000 *g* for 10 minutes, plasma was aliquoted and stored at −80°C. DNA was extracted using a TIANamp Blood DNA Kit (Tiangen Inc., Beijing, China). The rs7025417 and rs3821204 were genotyped by Taqman assay (Applied Biosystems, Foster City, CA, USA). The assay ID for the 2 SNPs was C_31940410_20 and C_1226153_10 respectively. The genotyping results were verified by Sanger sequencing and the concordant rate was 100%.

### 2.6 Real‐time PCR

Total RNA was isolated from peripheral blood cells using a commercial kit (Tiangen, Beijing, China). A total of 500 ng RNA was used for the reverse transcription reaction. A real‐time PCR assay was performed with a SYBR Premix Ex Taq on a gradient cycler (Mastercycler Gradient, Eppendorf, Germany). The sequences of oligonucleotide primers were as follows^22^, ^23^: *IL‐33* forward: 5′‐ATCCCAACAGAAGGCCAAAG‐3′ and reverse: 5′‐ CCAAAGGCAAAGCACTCCAC‐3′; *sST2* forward: 5′‐GGCACACCGTAAGACTAAGTA G‐3′ and reverse: 5′‐CAATTTAAGCAGCAGAGAAGCTCC‐3′; β*‐actin* forward: 5′‐ TTGCCGACAGGATGCAGAA‐3′ and reverse: 5′‐GCCGATCCACACGGAGTACT‐3′. Relative quantification of *IL‐33* mRNA to β*‐actin* was determined using a 2^−ΔCt^ calculation. Each assay was done in duplicate.

### 2.7 Plasma *IL‐33* levels

Plasma IL‐33 concentration was measured by an enzyme‐linked immunosorbent assay (Raybiotech, Norcross, GA, USA) according to the manufacturer's instructions. The minimum limit of IL‐33 detection was 2 pg/mL and the maximum limit was 500 pg/mL. All experiments were done in duplicate.

### 2.8 Plasmid construction, transfection and dual‐luciferase reporter gene assay

A 1956 bp promoter sequence of *IL‐33* containing the rs7025417 T allele was cloned into a pGL3 basic vector (Promega, Madison, WI, USA). After sequencing confirmation, the pGL3 vector containing the rs7025417 T allele was used as a template to yield a vector containing the rs7025417 C allele using the QuickChange Site‐Directed Mutagenesis kit (Stratagene, La Jolla, CA, USA). The insert sequences (rs7025417T and rs7025417C) were verified by Sanger sequencing. Plasmid containing the *ST2* rs3821204 G or rs3821204 C was constructed as described previously.^22^ Briefly, The 3′‐UTR fragment of *ST2* was amplified using the following primers: 5′‐GTCTCTAGAATCCCCCACTCCCTCC‐3′ (forward) and 5′‐CAGTCTAGAATCTGTGTTCCTGCCC‐3′ (reverse). The *ST2* rs3821204 G to C point mutation was introduced using the primers: 5′‐GTTTTTCTGGTCATAATGAAC‐3′ (forward) and 5′‐GTGTTCATTATGACCAGAAAAACGTATAGAACGG‐3′ (reverse). After digestion, the fragment was inserted into pGL3‐control vector (Promega).

The human OS cell lines (MG‐63 and Saos‐2) and the human embryonic kidney cell line 293 (HEK293) were cultured in medium supplemented with 10% foetal bovine serum. The cells were seeded into 24‐well plates and incubated at 37°C for 24 hours. The rs7025417T or rs7025417C plasmid (500 ng) was cotransfected simultaneously with 50 ng of a pRL‐TK vector (Promega) using Lipofectamine 3000 reagent (ThermoFisher Scientific, Waltham, MA, USA). Luciferase constructs containing *ST2* 3′‐UTR and miR‐202 mimics were cotransfected into HEK293 cells. After 48 hours, cells were lysed and firefly and renilla luciferase activity was measured with the Dual Luciferase Reporter Assay Kit (Promega).

### 2.9 Chromatin immunoprecipitation (ChIP) assay

The chromatin immunoprecipitation assay (ChIP) assay was performed with a commercial kit from ThermoFisher Scientific (Waltham, MA, USA) following the manufacturer's protocol. MG‐63 and Saos‐2 cells were cross‐linked in 1% formaldehyde for 10 minutes; DNA was then subjected to immunoprecipitation using antibodies against c‐Myb or non‐specific rabbit IgG (Abcam). Purified DNA was amplified by PCR with the primers: 5′‐TTGTGTCTCCTTTCCCCTACA‐3′ (forward) and 5′‐ATGCACACCAACCACTTTGA‐3′ (reverse). PCR product was then analysed by Sanger sequencing.

### 2.10 Statistical analysis

All statistical analyses were performed with the statistical software package SPSS 13.0 (SPSS Inc., Chicago, IL, USA). Continuous data, including age distribution and dual‐luciferase reporter gene activities, were compared using a Student's *t* test. HWE and the genotype distributions of the rs7025417 and rs3821204 in cases and controls were compared using chi‐squared test. Odds ratios (ORs) and 95% confidence intervals (CIs) were computed to assess the association of the rs7025417 and rs3821204 with OS risk. Differences in plasma IL‐33 levels in patients with OS and controls were analysed using a Mann‐Whitney rank‐sum test. Two‐sided *P* values <.05 were considered statistically significant.

## 3 RESULTS

### 3.1 Characteristics of the study subjects

The characteristics of controls and patients with OS are shown in Table 1. The median age of diagnosis in controls and cases was 18.0 (12.0‐61.0) and 17.0 (6.0‐61.0) years. No significant difference in age distributions was observed between controls and cases (*P *=* *.11). The gender distributions in both controls and cases were almost similar (*P *=* *.55). Most of the patients (71.9%) were diagnosed as tumours of long tubular bones and 70.9% patients had tumours without metastasis.

**Table 1:** Characteristics of controls and patients with osteosarcoma

Characteristics	Patients with osteosarcoma (n = 402)	Controls (n = 572)	*P* value
Age of diagnosis (median, y)	17.0 (6.0‐61.0)	18.0 (12.0‐61.0)	.11
Gender, n (%)			
Male	239 (59.5)	351 (61.4)	.55
Female	163 (40.5)	221 (38.6)	
Tumour location, n (%)			
Long tubular bones	289 (71.9)		
Axial skeleton	113 (28.1)		
Metastasis, n (%)			
Yes	117 (29.1)		
No	285 (70.9)		

### 3.2 Association of the *IL‐33/ST2* polymorphisms with OS risk

The distributions of the *IL‐33* rs7025417 and *ST2* rs3821204 in OS patients and controls are shown in Table 2. The genotype frequencies of the 2 SNPs were in Hardy‐Weinberg equilibrium (HWE) among cases and controls (*P *>* *.05). The rs7025417 CC genotype was significantly associated with a decreased risk of OS (CC vs TT: OR = 0.59, 95% CI, 0.41‐0.85, *P* = .005; dominant model: OR = 0.72, 95% CI, 0.55‐0.94, *P *=* *.02; recessive model: OR = 0.68, 95% CI, 0.49‐0.94, *P *=* *.02). Similarly, decreased risk of OS was also observed in allele comparison (OR = 0.76, 95% CI, 0.63‐0.91, *P *=* *.003). However, no evidence of association between the rs3821204 and OS risk was found. We then performed combined analyses to evaluate whether the combined genotypes of the 2 SNPs influenced the risk of OS. Compared to the rs7025417TT‐rs3821204CG/CC genotypes, the rs7025417CT/CC‐rs3821204CG/CC and the rs7025417CT/CC‐rs3821204GG genotypes were associated with a decreased risk of OS (OR = 0.61, 95% CI, 0.43‐0.87, *P *=* *.006; OR = 0.62, 95% CI, 0.43‐0.90, *P *=* *.01) (Table 3).

**Table 2:** Association between functional polymorphisms in *IL‐33/ST2* and the risk of osteosarcoma

SNPs	Controls, n = 572 (%)	Cases, n = 402 (%)	OR (95% CI)	*P* value
rs7025417				
TT	177 (30.9)	154 (38.3)	1.00 (Ref)	
CT	263 (46.0)	180 (44.8)	0.79 (0.59‐1.05)	.10
CC	132 (23.1)	68 (16.9)	0.59 (0.41‐0.85)	.005
Dominant	395 (69.1)	248 (61.7)	0.72 (0.55‐0.94)	.02
Recessive	440 (76.9)	334 (83.1)	0.68 (0.49‐0.94)	.02
T allele	617 (53.9)	488 (60.7)	1.00 (Ref)	
C allele	527 (46.1)	316 (39.3)	0.76 (0.63‐0.91)	.003
rs3821204				
GG	258 (45.1)	170 (42.3)	1.00 (Ref)	
CG	240 (42.0)	175 (43.5)	1.11 (0.84‐1.46)	.47
CC	74 (12.9)	57 (14.2)	1.17 (0.79‐1.74)	.44
Dominant	314 (54.9)	232 (57.7)	1.12 (0.87‐1.45)	.38
Recessive	498 (87.1)	345 (85.8)	1.11 (0.77‐1.61)	.58
G allele	756 (66.1)	515 (64.1)	1.00 (Ref)	
C allele	388 (33.9)	289 (35.9)	1.09 (0.91‐1.32)	.35

CI, confidence interval; OR, odds ratio; SNPs, single nucleotide polymorphisms.

**Table 3:** Combined analyses of the polymorphisms in *IL‐33/ST2* and the risk of osteosarcoma

SNPs	Controls (%)	Cases (%)	OR (95% CI)	*P* value
rs7025417TT‐ rs3821204CG/CC	92 (16.1)	94 (23.4)	1.00 (Ref)	
rs7025417TT‐ rs3821204GG	85 (14.9)	60 (14.9)	0.69 (0.45‐1.07)	.10
rs7025417CT/CC‐ rs3821204CG/CC	222 (38.8)	138 (34.3)	0.61 (0.43‐0.87)	.006
rs7025417CT/CC‐ rs3821204GG	173 (30.2)	110 (27.4)	0.62 (0.43‐0.90)	.01

CI, confidence interval; OR, odds ratio; SNPs, single nucleotide polymorphisms.

### 3.3 Association between the *IL‐33* rs7025417 and *IL‐33* mRNA and protein levels

**Figure 1:**
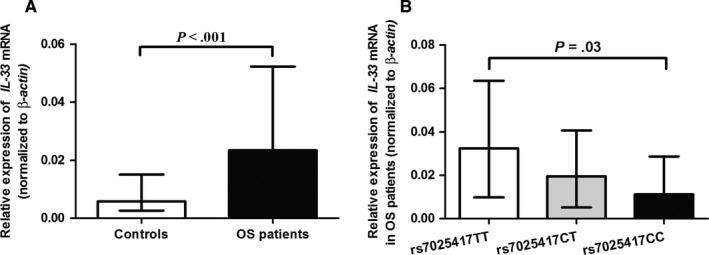
Box plots of *IL‐33* mRNA expression. Data were shown as median with interquatile range. A, Comparison of *IL‐33* mRNA levels in patients with osteosarcoma (OS) and controls. B, Comparison of *IL‐33* mRNA levels in patients with different rs7025417 genotypes

**Figure 2:**
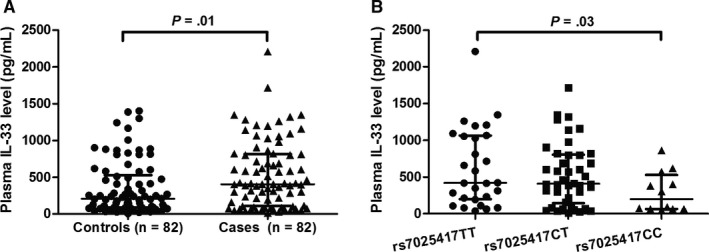
Scatter dot plots of IL‐33 protein levels. The lines inside the dots denote the median with interquatile range. A, Comparison of plasma levels of IL‐33 in patients with osteosarcoma (OS) and controls. B, Comparison of plasma levels of IL‐33 in patients with different rs7025417 genotypes

The relative expression of *IL‐33* mRNA was higher in OS patients than controls (*P *<* *.001, Figure 1A). We then analysed the correlation between the *IL‐33* rs7025417 and *IL‐33* mRNA levels. We found that the rs7025417 CC genotype was associated with a reduced *IL‐33* mRNA levels (*P* = .03, Figure 1B). Moreover, the median levels of plasma IL‐33 in OS patients was 405.3 pg/mL, which was higher than that in controls (206.9 pg/mL) (*P* = .01, Figure 2A). As shown in Figure 2B, patients with the rs7025417 CC genotype exhibited lower levels of IL‐33 compared to those with the TT genotype (*P *=* *.03).

### 3.4 Effect of the *IL‐33* rs7025417 on the transcriptional activity

**Figure 3:**
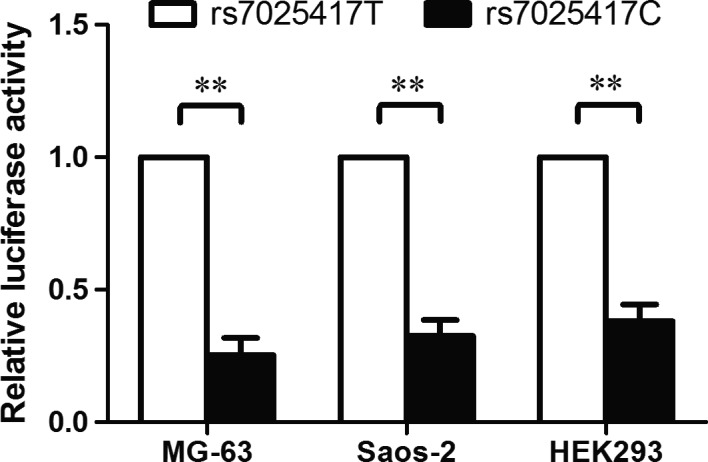
Dual‐luciferase reporter gene analysis. The human osteosarcoma (OS) cell lines (MG‐63 and Saos‐2) and the human embryonic kidney cell line 293 (HEK293) were transfected simultaneously with rs7025417T or rs7025417C plasmid and pRL‐TK vector. The luciferase activity was measured using the dual luciferase reporter assay. Data were expressed by mean ± standard deviation (**P <* *.01)

To identify whether the ‐1611 bp polymorphism (rs7025417) in the promoter of *IL‐33* influenced the transcriptional activity, we constructed plasmids containing rs7025417T and rs7025417C allele and analysed the luciferase activity. As shown in Figure 3, the rs7025417C allele presented a lower transcriptional activity compared to the rs7025417T allele in different cell lines, including MG‐63, Saos‐2, and HEK293, with *P* values of .004, .002, and .005 respectively.

### 3.5 *IL‐33* rs7025417 T allele‐specific binding to transcription factor c‐Myb

**Figure 4:**
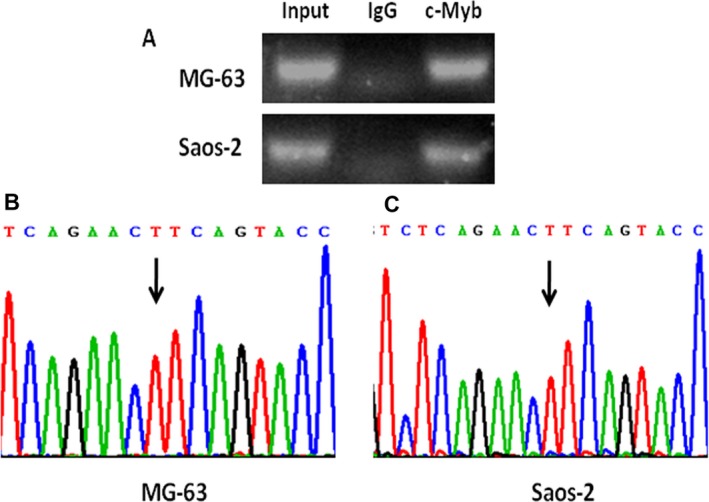
Transcription factor c‐Myb binds to the region containing rs7025417 TT genotype. ChIP assay was performed using anti‐c‐Myb antibody and anti‐IgG antibody as controls in MG‐63 and Saos‐2 cells (A). The presence of c‐Myb binding region containing rs7025417 TT genotype was verified by Sanger sequencing in MG‐63 (B) and Saos‐2 cells (C)

In silico analysis was used to predict allele‐specific transcription factor binding for rs7025417, and we found that rs7025417 T to C base change might affect the binding site of c‐Myb. Chip‐seq was then performed to assess the validity of allele‐specific transcription factor binding. As shown in Figure 4A, the DNA fractions were immunoprecipitated specifically with the c‐Myb antibody but not non‐specific IgG in MG‐63 and Saos‐2 cells. Further sequencing analysis revealed that the region contained rs7025417 TT genotype (Figure 4B,C).

### 3.6 The rs3821204 G/C influences the transcriptional activity and *ST2* mRNA expression by altering the binding site of miR‐202‐3p

**Figure 5:**
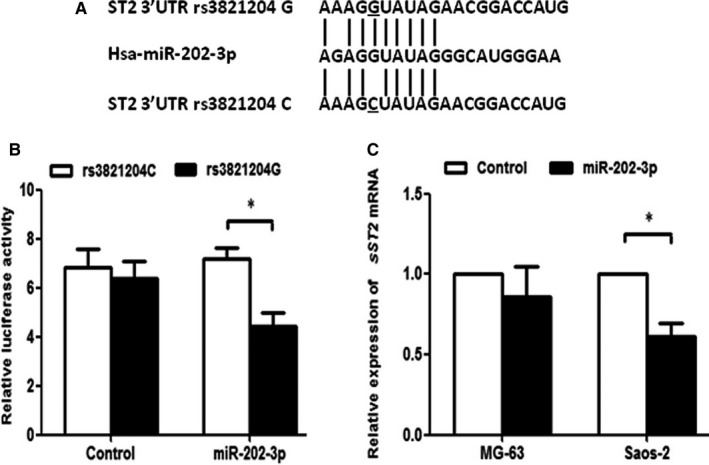
The rs3821204 G/C influences the transcriptional activity and *ST2* mRNA expression by altering the binding site of miR‐202‐3p. A, In silico prediction of miR‐203‐3p binding site to *ST2* 3′UTR containing rs3821204 G or C allele. B, The rs3821204 G or C plasmid was cotransfected with miR‐202‐3p mimics or negative control into HEK293 cells. Relative luciferase activity was measured using the dual luciferase reporter assay. C, miRNA‐202‐3p mimics or negative control was transfected into Saos‐2 (rs3821204 GG) and MG‐63 (rs3821204 CC) cells. Relative expression of *ST2* mRNA was analysed using real‐time PCR. Data were expressed by mean ± standard error (*P <* *.05)

Previous study showed that the rs3821204 disrupted a miR‐202‐3p seeding site, resulted in an allelic difference of *sST2* mRNA stability and expression, and finally modified the susceptibility of hypertension.^22^ In this study, we cotransfected rs3821204 G or C plasmid with miR‐202‐3p mimics (50 μmol/L) or negative control into HEK293 cells and confirmed the alteration of miR‐202‐3p binding site and a significantly decreased luciferase activity of rs3821204‐G but not rs3821204‐C (Figure 5A,B). Overexpression of miR‐202‐3p caused a decrease of *ST2* mRNA levels in Saos‐2 (rs3821204 GG) cells but not MG‐63 (rs3821204 CC) cells (Figure 5C).

## 4 DISCUSSION

The hospital‐based case‐control study showed that the rs7025417 CC genotype in the promoter of *IL‐33* had a decreased risk of OS in the Chinese population. Combined genotypes of the *IL‐33* rs7025417CT/CC‐*ST2* rs3821204CG/CC and the *IL‐33* rs7025417CT/CC‐*ST2* rs3821204GG also had a decreased risk of OS. The results from real‐time PCR and ELISA showed for the first time that *IL‐33* mRNA and protein levels in OS patients were higher than controls. Notably, *IL‐33* mRNA and protein levels in patients with the rs7025417 CC genotype were lower than those with the rs7025417 TT genotype. The dual‐luciferase reporter assay showed that the rs7025417C allele presented a lower luciferase activity. ChIP assay revealed that the rs7025417 T allele can specifically bind to transcription factor c‐Myb. Additionally, the rs3821204 G/C influences the transcriptional activity and *ST2* mRNA expression by altering the binding site of miR‐202‐3p. These findings suggest that the rs7025417 and rs3821204 may reduce the transcriptional activity and *IL‐33/ST2* expression, and eventually result in the decreased risk of OS.

A possible explanation for the positive results of the *IL‐33*CC genotype decreasing OS risk is that the rs7025417 located in the promoter region of *IL‐33* and exerted influences on the transcriptional activity. In this study, the dual‐luciferase gene reporter assay was performed and we found that the rs7025417 T to C shift decreased the luciferase activity. These findings were in agreement with previous results reported by Tu et al^21^ who found that the rs7025417 was significantly associated with the risk of coronary artery disease by regulating *IL‐33* gene expression. In addition to gene expression, we postulated that the rs7025417 may influence the protein level of IL‐33. Clinical studies investigating the expression of IL‐33 levels in cancer have yielded conflicting results. Some authors reported a highly expressed concentration of IL‐33 in serum and tumour tissues of ovarian cancer,^20^ breast cancer,^24^, ^25^ gastric cancer,^26^ non‐small cell lung cancer,^27^ colorectal cancer^28^ and squamous cell carcinoma of tongue.^29^ The higher IL‐33 expression predicted a worse prognosis and progression‐free survival after chemotherapy.^27^, ^29^, ^30^ Downregulation of IL‐33 expression can inhibit cell growth, metastasis and prolong the survival time.^28^ In contrast, some authors reported reduced IL‐33 levels in patients with multiple myeloma^31^ and lung cancer.^32^ No report to date examined the expression levels of IL‐33 in OS. In this study, we found that *IL‐33* mRNA and protein levels of were significantly increased in OS patients. Importantly, we found that *IL‐33* mRNA and protein levels were significantly lower in individuals with the rs7025417CC genotype than the homozygous TT genotype. Taken together, we may conclude that the rs7025417CC genotype had a protect effect on OS by reducing IL‐33 expression at both transcriptional and protein level.

It has been demonstrated that individual SNP may interact with each other in a biological manner if the interactions produce a more than multiplicative effect.^21^, ^33^ The IL‐33/ST2 pathway can regulate not only autoimmune and inflammatory conditions but also tumour growth and metastasis.^34^, ^35^, ^36^ By binding to ST2, IL‐33 can stimulate the production of immature dendritic cells that induce T‐regs, and thus facilitate tumour progression and metastasis.^36^ Deletion of IL‐33/ST2 axis can enhance cytotoxicity of NK cells, induce the secretion of interferon‐gamma, IL‐17 and tumour necrosis factor‐α, which attenuated tumour growth.^36^ These findings suggest the crosstalk of *IL‐33* and *ST2* in tumorigenesis. In this study, we found that the ORs of the combined genotypes of the 2 SNPs were lower than those of a single one in the association with OS. Further functional analysis revealed that the rs3821204 influences the transcriptional activity and *ST2* mRNA levels by altering the binding site of miR‐202‐3p. miR‐202‐3p was a tumour suppressor in several cancer types, such as gastric cancer^37^ and colorectal carcinoma,^38^ which may be used as a target to develop novel therapeutic approaches. Based on the results above, a conclusion was made that the *IL‐33* rs7025417 and *ST2* rs3821204 may affect the occurrence of OS by interaction with each other in a biological way.

We have to acknowledge some limitations of this study. As is well known, genetic variants may have different effect in diverse ethnicities. In the present study, only Chinese Han was recruited, and thus our results cannot be extended directly to other ethnic groups until confirmation analysis was performed. In addition to genetic factors, environmental factors are crucial in the development and progression of OS. The study design, however, did not take them into consideration, which prevented our further interaction analysis of *IL‐33* and environmental factors with the risk of OS.

In conclusion, we found a functional polymorphism in the promoter of IL‐33 (rs7025417). The rs7025417 CC genotype protected against OS tumorigenesis and reduced IL‐33 expression levels by decreasing the transcriptional activity. Our findings further supported the key role of *IL‐33/ST2* pathway in OS and highlighted *IL‐33/ST2* as valuable targets for prevention and treatment of OS.

## CONFLICT OF INTEREST

None declared.

## AUTHOR CONTRIBUTION

Jun‐Li Wang and Yu‐Jin Tang designed and wrote the manuscript. Jia Liu collected samples. Jun‐Li Wang and Jia Liu performed experiments and data acquisition. Ke‐Gong Xie performed statistical analysis. Chang‐Gong Lan and Lu Lu prepared figures. All authors reviewed the manuscript.
